# Prevalence of Suicidal Behavior and Associated Clinical Correlates in Patients with Behavioral Addictions

**DOI:** 10.3390/ijerph182111085

**Published:** 2021-10-21

**Authors:** Eduardo Valenciano-Mendoza, Fernando Fernández-Aranda, Roser Granero, Mónica Gómez-Peña, Laura Moragas, Bernat Mora-Maltas, Anders Håkansson, José M. Menchón, Susana Jiménez-Murcia

**Affiliations:** 1 Department of Psychiatry, University Hospital of Bellvitge-IDIBELL, 08907 Barcelona, Spain; edevalenciano@idibell.cat (E.V.-M.); ffernandez@bellvitgehospital.cat (F.F.-A.); monicagomez@bellvitgehospital.cat (M.G.-P.); lmoragas@bellvitgehospital.cat (L.M.); bmora@idibell.cat (B.M.-M.); jmenchon@bellvitgehospital.cat (J.M.M.); 2 CIBER Fisiopatología Obesidad y Nutrición (CIBERObn), Instituto de Salud Carlos III, 28029 Madrid, Spain; Roser.Granero@uab.cat; 3 Department of Clinical Sciences, School of Medicine and Health Sciences, University of Barcelona, 08907 Barcelona, Spain; 4 Department of Psychobiology and Methodology, Autonomous University of Barcelona, 08193 Barcelona, Spain; 5 Department of Clinical Sciences Lund, Psychiatry, Faculty of Medicine, Lund University, 22 185 Lund, Sweden; anders_c.hakansson@med.lu.se; 6 Region Skåne, Malmö Addiction Center, Gambling Disorder Unit, 205 02 Malmö, Sweden; 7 CIBER Salud Mental (CIBERSAM), Instituto de Salud Carlos III, 28029 Madrid, Spain

**Keywords:** suicide, behavioral addiction, sociodemographic factors, personality traits, psychopathology

## Abstract

Addictive disorders are characterized by severe consequences, including suicidal events, but most studies investigating the association between addiction and suicidal risk have focused on substance use disorders and gambling disorder at the expense of the rest of behavioral addictions. This study examined the prevalence and the associated clinical correlates of suicidal ideation and suicide attempts in a sample of patients with a diagnosis of behavioral addiction. The total sample consisted of 4404 individuals: 4103 of these patients with gambling disorder, 99 with gaming disorder, 44 with sex addiction, and 158 with buying–shopping disorder. All of them were assessed consecutively at a specialized hospital unit for the treatment of behavioral addictions. Participants attended two clinical interviews and completed self-reported questionnaires to explore clinical features of behavioral addictions, personality traits, psychopathological symptomatology, suicidal behavior, and sociodemographic variables. The highest prevalence of suicidal ideation was found in patients with gambling disorder (22.9%), followed by buying–shopping disorder (18.4%), sex addiction (18.2%), and gaming disorder (6.1%). The highest prevalence of suicide attempts was registered for sex addiction (9.1%), followed by buying–shopping disorder (7.6%), gambling disorder (6.7%), and gaming disorder (3.0%). Female gender and unemployment constituted two relevant sociodemographic factors associated with suicidal risk in gambling disorder, gaming disorder, and buying–shopping disorder. Lack of family support appeared as a relevant risk factor, except for gaming disorder. These results pointed out that suicide is a prevalent behavior in behavioral addictions, and clinicians and researchers need to pay particular attention to the specificities of each behavioral addiction when assessing suicidal risk.

## 1. Introduction

At present, there is broad evidence to sustain that addictions are not only limited to behaviors related to substance use, but that there are a series of behavioral habits that can become addictive and seriously interfere with the individual’s life [1,2,3]. Nevertheless, there is still an ongoing debate regarding the existence and the conceptualization of behavioral addictions as mental disorders [1,4]. While several authors postulate the entity and utility of this construct [5,6,7], others consider it premature to apply this term to a wide range of clinical entities [8,9,10,11]. These different ways of conceptualizing these maladaptive behaviors have led to the development of different diagnostic approaches to these behaviors. Therefore, except in the case of gambling disorder, which has been largely investigated during the last decades [12], there is no consensus on the diagnostic criteria, thresholds, and assessment tools for the rest of behavioral addictions and further research is needed to improve knowledge on these conditions [4]. Despite the fact that we acknowledge the controversy regarding the concept of behavioral addiction, this term will be used along this article to embrace this set of clinical entities.

Behavioral addictions (also known as non-substance addictions) and other mental disorders such as affective, anxiety, and substance use disorders, as well as suicidal behavior may co-occur [13,14,15,16,17,18,19]. While in the case of substance use disorders there is large literature on the risk of suicide depending on the substance consumed [20], in the case of behavioral addictions the available evidence is very uneven depending on the subtype of behavioral addiction. Most studies that have investigated the relationship between behavioral addiction and suicidal behavior have focused on gambling disorder [21]. In contrast, other addictive behaviors such as gaming disorder, sex addiction, or buying–shopping disorder have hardly been investigated in their relationship with suicide risk. This gap in the literature may be due to the fact that gambling disorder was recognized as a mental disorder in the 1980s (first as an impulse control disorder and later as a behavioral addiction) [22] while the other addictive behaviors have not been formally recognized as such, except for gaming disorder which has recently been recognized as an addictive disorder in the 11th edition of the International Classification of Diseases (ICD-11) [23,24]. In the last edition of the International Classification of Diseases (ICD-11), compulsive sexual behavior disorder has been included as an impulse control disorder and buying–shopping disorder is mentioned as an example for “other specified impulse control disorders” [24].

Several characteristics have been described that resemble behavioral addictions to substance use disorders, including short-term reward, the loss of control over the behavior so that it is carried out despite the serious consequences, the growing need to increase the intensity and frequency of the behavior to experience the same level of initial arousal (tolerance), and the physical and cognitive symptoms that appear when the behavior is interrupted (withdrawal syndrome) [25,26,27].

The fact that people with addictive disorders are more prone to suicidal behavior has been attributed to the observation that such behavior is often driven by impulsivity [28] and that addictive disorders are characterized precisely by high levels of impulsivity, as well as difficulties in inhibitory control [29,30,31]. In a recent systematic review and meta-analysis, the authors identified that the highest risk of suicidal ideation in individuals with substance use disorders were associated with smoking, history of sexual abuse, depressive symptomatology, and alcohol and cannabis use disorders. Regarding risk factors associated with suicide attempts, the authors identified that being female, having a history of sexual abuse, depressive symptomatology, and the presence of alcohol, cannabis, cocaine, or amphetamine use disorders, as well as polysubstance abuse, were the most reported factors [20].

In the case of gambling disorder, there are several factors that appear to be often described as associated with suicidal behavior. These factors include being female [32,33], depressive symptoms [34], having financial debts or facing bankruptcy [35], unemployment [36], lack of family support [33,37], greater severity of the gambling disorder [38], and worse psychopathological state [32,39]. In addition, higher levels of novelty seeking and harm avoidance were also identified as personality traits associated with suicidal risk [34].

Most studies that focused on analyzing the association between gaming disorder and suicidal behavior have been carried out in Asian countries. This fact is explained by the high prevalence of this disorder in those countries, although it is a growing phenomenon in other parts of the world [40,41,42]. In a study carried out in China, depressive symptoms, insomnia, female gender, lower social class, and younger age were described as sociodemographic risk factors for suicidal ideation [43]. Another study pointed out the possible link between playing violent video games and suicidal behavior, through the progressive exposure to visualizing pain-related situations and thus increasing tolerance to pain and risk taking behavior. It has been hypothesized that these factors may contribute to enhance individual’s capability for suicide [44]. On the contrary, a recent study found that the association between internet gaming disorder and suicidal behavior was no longer significant after adjusting for the presence of comorbid psychiatric disorders and poor health status [15]. While the literature on the association between gaming disorder and suicidal behavior is scarce, more studies have been carried out to assess the relationship between internet addiction and suicide. Internet addiction covers a wide range of problematic addictive behaviors related to the use of the internet including internet gaming addiction [45]. On a meta-analysis, the authors concluded that suicidal ideation and suicide attempts rates were still elevated in individuals with internet addiction, even after adjusting for the effects of depression, and postulated the need to further explore other relevant factors that could explain the association between internet addiction and suicide [46].

The literature on the association between sex addiction and suicidal behavior is surprisingly limited. Chatzittofis et al. found that men with hypersexual disorder and a history of suicide attempts reported higher odds of having experienced childhood adversity (including sexual abuse) and exposure to violence as an adult [47]. In another study, the authors posited that non-paraphilic problematic sexual behavior was associated with impulsivity, emotion dysregulation, problematic use of the internet, current suicidal behavior, anxiety and depression symptomatology, and low self-esteem [48]. However, Shirk et al. did not find a significant association between problematic pornography use and suicidal ideation [49].

Regarding buying–shopping disorder, a recent study described that problematic shopping and non-suicidal self-injury were associated with greater levels of impulsivity and sensation-seeking, although no association between suicidal behavior and problematic shopping were assessed [18].

Gaming disorder, gambling disorder, sex addiction, and buying–shopping disorder are characterized by a loss of control [18,50]. In the last three addictions, this symptomatology is accompanied with feelings of shame and guilt for the severe consequences of the behavior (financial difficulties, lies, and even criminal behavior), negative social perception of these addictive behaviors, and addictive behavior-related harm cause to relatives and friends [51,52,53,54]. These feelings have been identified as relevant predictors for suicidal behavior [55,56].

The primary aim of the present study was to examine and compare the prevalence of suicidal ideation and history of suicide attempts in large sample of patients with a diagnosis of behavioral addiction. The second aim was to investigate the clinical and personality correlates associated with suicidal behavior in patients with behavioral addictions.

In terms of prevalence, it was hypothesized that those disorders characterized by higher levels of feelings of shame and guilt (that is, gambling disorder, sex addiction, and buying–shopping disorder) will show greater prevalence of suicidal behavior. Based on the previous literature, it was also hypothesized that a set of transdiagnostic clinical correlates will be present in the four behavioral addictions: female gender, unemployment, lack of family support, and depressive symptomatology.

## 2. Methods

### 2.1. Sample

The total sample consisted of 4404 individuals of whom 4103 patients with gambling disorder, 99 with gaming disorder, 44 with sex addiction, and 158 with buying–shopping disorder. All the participants were consecutively assessed at the behavioral addictions unit at Bellvitge University Hospital (Barcelona, Spain) between January 2005 and May 2020. Consecutively admitted patients were derived to the unit through general practitioners or via another healthcare professional. The following exclusion criteria were established: (a) the presence of an organic medical condition such as a neurocognitive disease, and (b) lifetime history of brain injury or intellectual disabilities.

### 2.2. Procedure

Upon arrival to the treatment unit, all patients were informed about the research character of the treatment unit, and they were asked to give their consent for incorporating their assessments within the group analyses guaranteeing the total anonymity (this is a common procedure performed in secondary and tertiary care centers in our country). All patients provided signed consent, and exclusions were based on inclusion–exclusion criteria.

During a first clinical interview conducted by an experienced clinical psychologist or psychiatrist, participants meeting criteria for one of the behavioral addictions considered (gambling disorder, gaming disorder, sex addiction, or buying–shopping disorder) were invited to take part in the study. As part of the assessment process, all participants attended an additional clinical interview and completed a set of questionnaires to collect clinical and socio-demographic information under the supervision of experienced psychologists. Clinicians in charge of collecting this information were doing so as part of a standard screening process within the behavioral addictions unit and they were not specifically informed about the objectives of the study. Therefore, specific clinicians’ expectations when assessing patients did not influence the result of the present study. All clinical information was directly provided by participants in the study and no third-party reports from friends or relatives were used. Participants did not receive any compensation for being part of the study.

### 2.3. Measures

#### 2.3.1. Semi-Structured Clinical Interview

The presence of a behavioral addiction was assessed through a semi-structured face-to-face clinical interview conducted by a clinical psychologist or psychiatrist with more than 20 years of experience in the diagnosis of behavioral addictions. Diagnostic criteria for gambling disorder and gaming disorder were based on DSM-5 criteria. While gambling disorder is formally recognized as a mental disorder according to DSM-5, gaming disorder has not received this recognition in DSM-5 yet due to insufficient evidence available. It is currently defined as a condition that requires further investigation to be considered as a behavioral addiction. For cases assessed between 2005 and 2013 (i.e., before the releasing of DSM-5), gaming disorder was assessed through a semi-structured interview adapted from DSM-III-R pathological gambling criteria [57]. Diagnostic criteria for buying–shopping disorder were determined following guidelines established by McElroy et al. [58], which have been widely accepted by the scientific community in spite of the fact that their reliability and validity are still not determined [59]. Regarding sex addiction, a list of items was used. These items were based on the consensual definition in the DSM-IV-TR [60] in the Sexual Disorders Not Otherwise Specified section (302.9).

The diagnostic status for the behavioral addictions (present versus absent, for gambling disorder, gaming disorder, sex addiction, and buying–shopping disorder) was based on the standardized diagnostic measurement tools, which used an algorithm based on the DSM criteria (the specific threshold and cut-off points defined in this taxonomy were employed).

During the clinical interview, information concerning the onset and duration of the disorder, debts due to behavioral addiction and perceived family support was also collected. Information on suicidal behavior (including suicide ideation and attempts) was collected through the following questions: (1) Have you ever had in the past or do you currently have thoughts related to death or the desire to die? (2) Have you ever attempted to take your own life? Face-to-face clinical interviews have been reported to be a valid tool to assess suicidal behavior [61].

#### 2.3.2. Self-Reported Measures

Based on the aims of the current study, all participants completed self-reported questionnaires to explore psychopathological symptoms, personality traits, sociodemographic and other relevant clinical variables.

Symptom Checklist-Revised (SCL-90-R) [62]. This self-reported questionnaire was created to assess the psychological state based on 90 items factorized into nine primary (first order) dimensions (somatization, obsessive-compulsive, interpersonal sensitivity, depression, anxiety, hostility, phobic anxiety, paranoid ideation, and psychoticism), and three global indices (global severity index (GSI), positive symptom total (PST), and positive symptoms discomfort index (PSDI)). The psychometric Spanish adaptation of this tool is available and has obtained adequate properties (the mean Cronbach’s alpha was α = 0.75) [63]. In the present study’s sample, the internal consistency was also in the adequate to good range (α = 0.789, for the paranoid ideation scale, to α = 0.981 for the global indices).

Temperament and Character Inventory-Revised (TCI-R) [64]. This is a 240-item questionnaire -which has been adapted for Spanish population obtaining adequate properties (the mean Cronbach’s alpha was α = 0.87) [65]—that measures four dimensions of temperament (novelty seeking, harm avoidance, reward dependence, and persistence) and three related to character (self-directedness, cooperativeness, and self-transcendence). In this study, the internal consistency in the sample was in the adequate to good range (α = 0.702, for novelty seeking, to α = 0.886 for persistence).

Sociodemographic information and other clinical variables: Socio-demographic characteristics including gender, age, marital status, education level, employment status, socio-economic position index—according to Hollingshead’s scale which is based on the participants’ level of education and profession [66]—was collected through a self-reported questionnaire.

### 2.4. Statistical Analysis

Statistical analysis was carried out with Stata16 (Stata-Corp, Texas, USA) for Windows [67]. The estimation of the prevalence of suicidal behavior was obtained for each diagnostic subtype, and the comparison between the groups was based on odds ratio coefficients (OR) calculated in logistic regression (effect size was considered medium–moderate for OR > 1.50, good for OR > 2.0, and large–high for OR > 3.00) [68].

The comparisons between the groups (defined for the diagnostic subtypes or the presence–absence of suicidal behavior) were based on chi-square tests (χ^2^) for categorical variables and analysis of variance (ANOVA) for quantitative measures. For the psychopathological state (SCL-90R) and the personality traits (TCI-R), the comparison between the groups with and without suicidal behavior was added as covariates the participants’ sex and age (ANCOVA), to avoid bias due the potential confusing role of these features.

In this study, Finner’s method (a familywise error rate—FWER—procedure which is more powerful than the classical Bonferroni correction) was used to control the increase in Type-I error due to multiple statistical tests [69]. When using the Finner correction, a fixed number of k-1 of erroneous rejections is tolerated, and where all the null hypotheses are equal, controlling the FWER at level α is equivalent to the problem of combining *p*-values to obtain a single testing for the null-hypothesis which is at level α. In practice, this method is employed by adjusting the rejection criteria for each of the individual hypothesis fixing the FWER rate no higher than a certain prespecified significance level α. In this study, the correction was done for groups of tests: sociodemographics, behavioral addiction related measures (onset, duration, and comorbid problems measured with the SCL-90R), and personality traits.

The effect size for the mean differences in the ANOVA and ANCOVA procedures was measured using the standardized Cohen’s-*d* coefficient (effect size was considered low–poor |*d*| > 0.20, moderate–medium for |*d*| > 0.5, and large–high for |*d*| > 0.8) [70,71].

## 3. Results

### 3.1. Characteristics of the Participants

Table 1 displays the description for the sociodemographic and the clinical variables of the study, stratified by the diagnostic subtype. Statistical differences between the groups were obtained for all these variables. Patients with gambling disorder included mainly men (91.2%), married (45.7%), in a low social position index (51.7%), primary education level (58.5%), and employed (56.2%). Patients with gambling disorder mean age was 42.5 years old (SD = 13.8) and mean age onset 30.2 (SD = 12.1). As regards the gaming disorder, most participants were men (92.9%), single (90.9%), in a low social position index (56.6%), primary education level (49.5%), and unemployed (79.8%). For gaming disorder, mean age was 23.0 years old (SD = 9.7) and mean age onset was 18.4 (SD = 8.7). In the sex addiction subtype, most participants were men (97.7%), married (54.5%), within mean-low social position index (34.1%), secondary education level (43.2%), and employed (70.5%). For sex addiction, mean age was 43.1 years old (SD = 11.8) and mean age onset 32.9 (SD = 12.8). Regarding buying–shopping disorder, most participants were women (74.7%), married (45.6%), within low social position index (41.8%), secondary education level (40.5%), and unemployed (52.5%). Among buying–shopping disorder, mean age was 43.4 years old (SD = 11.5) and mean age onset was 33.8 (SD = 12.7).

#### 3.1.1. Prevalence of Suicidal Behavior in the Study

**Figure 1:**
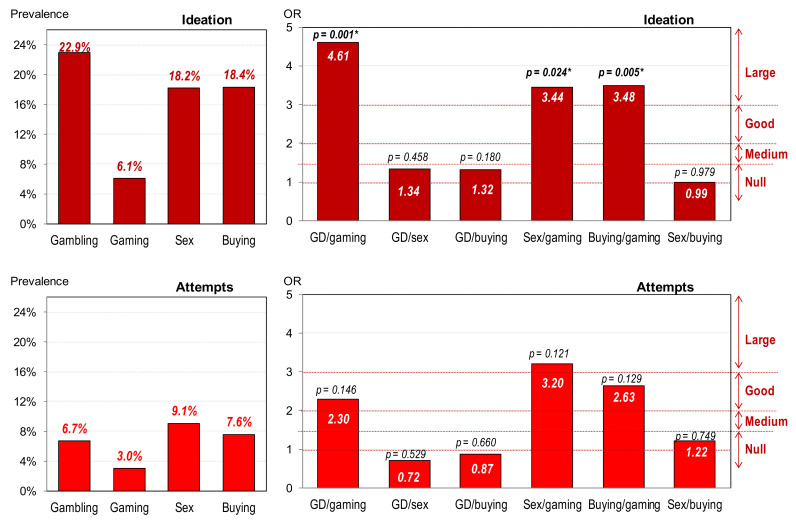
Prevalence of suicidal behavior and comparison between groups. * Bold: significant comparison (0.05).

Figure 1 displays the bar-charts with the prevalence of suicidal behavior in the study (ideation and attempts), as well as the results of the pairwise comparisons between the diagnostic subtypes (OR and *p*-values). The presence of suicidal ideation achieved the highest prevalence in the patients with gambling disorder (22.9%), nearly followed by buying–shopping disorder (18.4%) and sex addiction (18.2%), while the prevalence for gaming disorder was the lowest (6.1%). Regarding the presence of suicide attempts, the highest prevalence was registered for sex addiction (9.1%), followed by buying–shopping disorder (7.6%) and gambling disorder (6.7%), and the lowest prevalence was obtained for gaming disorder (3.0%). Although there were no statistical differences in the post-hoc comparisons for the presence of suicide attempts between the diagnostic subtypes, OR coefficients were within the good to large range for the contrasts between gaming disorder and the other groups.

#### 3.1.2. Comparison between Patients with and without Suicidal Behavior

**Figure 2:**
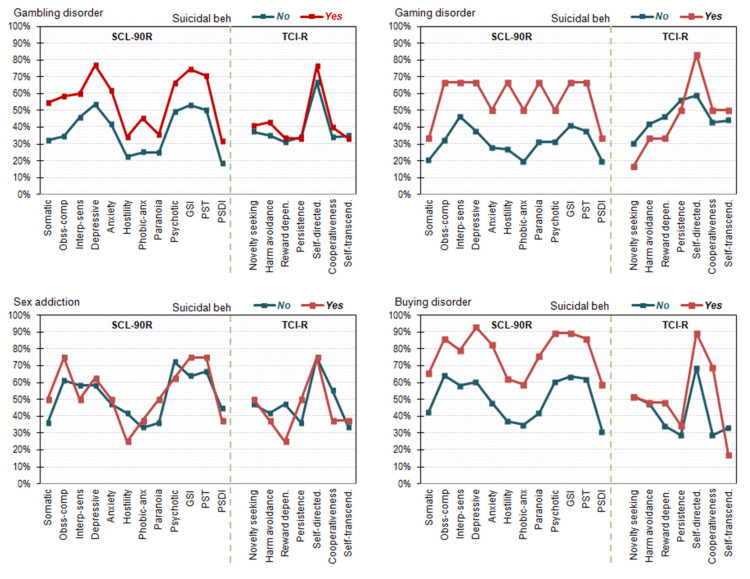
Prevalence of patients outside the normative ranges in the psychopathology and personality.

Table 2 shows the comparison between patients with and without suicidal behavior for the sociodemographic and the clinical profiles (Figure 2 shows the line-plots with the prevalence of patients outside the normative ranges in the SCL-90 and TCI-R scales). These analyses were carried out stratified (separately) for the diagnostic subtype, and suicidal behavior was considered for patients who reported ideation or attempts. For the comparisons within the diagnostic subsamples of gaming disorder, sex addiction, and buying–shopping disorder, the contingency variables that did not meet the condition of high expected frequencies (e_ij_ ≥ 5) were analyzed with exact chi-square tests. In addition, quantitative variables did not show significant deviations from Normality within the groups with low sample size (the Shapiro–Wilk test was used). In addition, and based on the low sample size of participants who reported suicidal behavior for gaming and sex addiction, differences between the groups were considered relevant for statistical significance or effect size from moderate to large ranges. Among patients with gambling disorder, the presence of suicidal behavior was related to female gender, being divorced or separated, unemployed occupational status, older age, longer duration of the disorder, lack of family support, presence of accumulated debts due to the gambling activity, worse psychopathological state, and more dysfunctional personality traits. As regards patients with gaming disorder, suicidal behavior likelihood was increased for female gender, lower social position indexes, unemployment status, higher scores in the SCL-90R obsessive-compulsive, anxiety, hostility, and global indexes (GSI and PST), lower score in the self-directedness trait, and higher score in the self-transcendence trait. For sex addiction, the likelihood of suicidal behavior was increased for divorced/separated patients, those in a lower social position indexes, shorter duration of the addictive problems, without family support, higher self-directedness, and lower self-transcendence. Finally, regarding the buying–shopping disorder, the presence of suicidal behavior was higher for women, patients in lower social indexes, unemployed, without family support, worse psychopathological state, and lower scores in the self-directedness and cooperativeness scales.

## 4. Discussion

Previous studies have reported high rates of suicidal behavior in substance use disorders [20,72] and gambling disorder [14,51,73], while little is known regarding the remaining behavioral addictions. The present study aimed at examining the prevalence of suicidal ideation and suicide attempts in a sample of patients with a diagnosis of a behavioral addiction, and identifying the clinical correlates associated with suicidal behavior.

### 4.1. Prevalence of Suicidal Behavior in Behavioral Addictions

The study’s findings indicated that patients at greater risk of committing a suicidal event (either suicidal ideation or attempts) are those with gambling disorder, sex addiction, or buying–shopping disorder. This result confirmed the first hypothesis, according to which these disorders would show greater rates of suicidal behavior compared to gaming disorder. Following this first hypothesis, the fact that gambling disorder, sex addiction, and buying–shopping disorder are characterized by higher levels of feelings of shame, isolation, and guilt [51,52,53,74] may contribute to increase the risk of suicide. Even though suicide is a complex multi-factorial phenomenon [75], there is large literature pointing out that feelings of shame and guilt are relevant drives for committing a suicidal act [55,56]. In addition, stigma has been reported to be particularly predominant in these disorders [76,77,78] and the former has been described as a trigger for suicidality [79]. The high prevalence of suicidal behavior in these three disorders constitute a relevant finding from the present study. In this regard, it is relevant to mention that diagnosis can be a double-edged sword: on the one hand, it facilitates communication among professionals and enables the development of more specific treatments. On the other hand, being diagnosed with a mental disorder (such as gaming disorder) may carry a certain degree of social stigma and this can increase feelings of shame and guilt [80] which, in the end, can be triggers for suicidal behavior [55].

While this study indicated that patients with gambling disorder are those with greater risk of showing suicidal thoughts, the highest prevalence of suicide attempts was found in sex addiction and buying–shopping disorder. A possible explanation to account for the highest likelihood of suicide attempts in sex addiction and buying–shopping disorder may be that participants in the present sample showed a worse psychopathological state and the last has been reported as a relevant contributor to suicide attempts [81]. This finding pointed out the greater severity of these two disorders in terms of suicidal risk. In a sample of hypersexual men, the authors found that 12% reported suicide attempts [47] which is a prevalence rate similar to the one found in the present study (9.1%). Unfortunately, the literature on suicide in these two disorders is very limited and therefore, it constitutes a priority to carry out further research on this topic.

Even though the risk of suicide in gaming disorder found in the present study is significantly low compared to the rest of behavioral addictions examined, the existence of this risk underlines the need to further explore clinical correlates of this dramatic behavior in this profile of patients, especially bearing in mind that adolescents are at particular risk to be affected by this disorder [82]. Lifetime prevalence of suicidal ideation and suicide attempts in the general population in Spain has been estimated to be 3.67% and 1.46% respectively [83]. Therefore, prevalence of suicidal behavior among individuals with gaming disorder found in the present study is almost twice as high compared to the general population.

From a clinical point of view, it could be added that, in general terms, while patients with gambling disorder, sex addiction, or buying–shopping disorder usually present greater awareness of illness and motivation to change (because of the severe and negative consequences they experience derived from their addictive disorder), in the case of patients with gaming disorder, this is not always the case. Mostly, they ask for treatment forced by their environment (families, partners, ...), showing resistance to psychological intervention, and to accept that they have problems with the use of video games and negative consequences at all levels. Thus, it is common to identify pre-contemplative states in patients with gaming addiction. In addition, patients with gaming disorder are generally younger compared to patients with other behavioral addiction and preserve a strong family support [41], and the last has been reported as a protective factor to suicidality [84].

### 4.2. Clinical Correlates Associated with Suicidal Behavior

A better knowledge of the clinical correlates that appear associated with suicidal behavior in behavioral addictions may contribute to identify patients at special risk and may help to develop interventions to reduce it. From a comparative approach, the authors of the present study tried to identify certain variables that may be triggers for suicidal events in all four behavioral addictions, paying particular attention to the influence of female gender, unemployment, lack of family support, and depressive symptomatology as the most promising factors.

Regarding female gender, findings from the study indicated that women are at higher risk in all behavioral addictions except in sex addiction. The fact that women are a vulnerable group in terms of suicide has been previously reported in other studies. For example, several studies carry out in a sample of treatment-seeking patients with gambling disorder have also reported female gender as a risk factor [32,33,85] and gender disparities in suicide rate may be explained by differences in emotional and behavioral problems [86]. In gaming disorder, women have also been identified as a vulnerable group in terms of suicidal behavior [43]. It is important to note that women may be at risk of reporting higher rates of suicidal behavior in the case sex addiction, but the almost absence of women in the subsample of patients with this disorder made it impossible to detect the potential risk. The existing discrepancy between the prevalence of women with sex addiction in the general population and in clinical setting highlights the greater stigma that women with this disorder suffer and the need for developing special programs to tackle this hidden reality [87].

Unemployment has also been reported as a risk factor for suicide in the case of gambling disorder [35,88,89], in other mental disorders including major depressive disorder [90] and bulimia nervosa [91,92,93], as well as in the general population irrespectively of the presence of a mental disorder [94,95]. Findings from the present study confirmed the relevance of unemployment in all behavioral addictions except for sex addiction. Financial problems, social isolation, and feelings of failure that frequently come with unemployment may contribute to the increase of hopelessness [96] and, consequently, the risk of suicide [97]. Further studies with larger samples of patient with sex addiction are needed to examine whether unemployment would play a role in this disorder.

Another relevant finding from the present study is the role of family support in individuals with behavioral addiction and suicidal behavior. The results indicated that the lack of family support contributed to increase the presence of suicidal behavior in patients with gambling disorder, sex addiction, and buying–shopping disorder. It has been hypothesized that in the case of gaming disorder—particularly in internet gaming—the community of gamers may work as a mutual support group [98]. Following this idea, online communities of gamers may be to some extent beneficial, especially in those individuals with great difficulties to make relationships.

Unexpectedly, worse psychopathological state and, in particular, depressive symptomatology, only appeared to be associated with suicidal behavior in gambling and buying–shopping disorder. This finding is somehow unanticipated given that depressive symptomatology has been systematically identified as a risk factor for suicide in addictive disorders [34,46,48] and in other mental disorders [99].

In terms of personality, only self-directedness dimension appeared to be associated with suicidal behavior in all behavioral addictions although in the opposite direction in the case of sex addiction. Previous studies have reported lower levels of self-directedness—that is, lack of a tendency to be goal-oriented and self-confident—as a risk factor for suicidal behavior in different mental disorders, including major depressive disorder, bipolar disorder [100], and eating disorders [101,102]. The present study’s findings replicate the relevance of low self-directedness as a clinical correlated associated with suicide in behavioral addictions. Patients with sex addiction showed higher socio-economic and educational levels and this profile of individuals are more prone to present higher levels of self-directedness [103,104]. Nevertheless, more studies are needed to better understand the role of personality and suicidality in patients with sex addiction.

Surprisingly, in the present study financial debts only appeared to be associated with suicide in gambling disorder, while in the rest of behavioral addictions did not. A possible explanation for this finding is that gamblers continue to gamble to recuperate losses, which causes them to enter into an uncontrolled loss spiral [105] and this phenomenon is not observed in the rest of behavioral addictions.

Finally, it is also important to highlight that certain clinical correlates of suicidal behavior are exclusively related to a particular behavioral addiction. For example, longer duration of the disorder only appeared to be relevant in gambling disorder in accordance with previous literature on other mental disorders such as eating disorders [91,106,107]. These specificities of each disorder call for the need to pay particular attention to the clinical particularities associated with each disorder when assessing patients with behavioral addictions at risk of committing a suicidal act.

### 4.3. Limitations and Strengths

First, the subgroup of patients with suicidal behavior in gaming disorder and in sex addiction was very small, limiting the possibility of drawing solid conclusions regarding these two disorders. In addition, it is reasonable to assume that people’s knowledge about the existence and treatment options of behavioral addictions varies substantially across conditions. While most people may know the existence of gambling disorder, this may not be the case for the rest of behavioral addictions. Therefore, this fact may contribute to exacerbate selection effects in the sample and may be altering differences between groups (e.g., very few individuals with buying–shopping disorder may know that this condition exists and hence a very reduced group of these individuals seek treatment). Second, assessment of suicidal behavior was exclusively based on a clinical interview. It would have been more informative to complement this information with self-reported questionnaires to measure the presence of suicidal thoughts and antecedents of suicide attempts. Third, this study has a cross-sectional design and therefore there is no possibility to establish causality between clinical correlates and the risk of suicide. Fourth, the possible link between feelings of stigma and shame, and an increased risk of suicidality in gambling disorder, buying–shopping disorder, and sex addiction has been inferred rather than directly tested in the study. Future research may consider assessing these variables to empirically test this hypothesis. Finally, it is worth mentioning that data from the present study was directly obtained from participants. However, self-reported information entails a response bias [108]. Therefore, it could be interesting for future research to include other methodologies in order to collect data that avoid the subjectivity bias. A possible alternative could be bank records that benefit from greater objectivity and data can be collected from very large groups of population [109]. These alternative methodologies could be of special interest in the case of those behavioral addictions that are underrepresented in clinical settings.

Nevertheless, the present study also has relevant strengths. First, there is a large sample size of patients with gambling and buying–shopping disorder. Second, to the authors’ knowledge this is the first study examining the prevalence of suicidal ideation and attempts in a sample of patients with the most relevant behavioral addiction following a comparative approach.

## 5. Conclusions

The present study contributed to highlight that suicide is a prevalent phenomenon in behavioral addictions. While several studies have explored this association in gambling disorder, the literature on the remaining behavioral addictions is very scarce. Patients with gambling disorder, sex addiction, and buying–shopping disorder are at special risk of suicidal ideation and attempts. No transdiagnostic clinical correlates of suicidal behavior have been found in all behavioral addictions, but female gender, unemployment, and lack of family support constitute three relevant factors present in most of the examined disorders. Although gaming disorder showed the lowest rates of suicide, the fact of being a condition that particularly affects adolescents makes it an issue of concern. Given the clinical specificities of each behavioral addiction, these results seem to indicate that clinicians and researchers need to pay special attention to the particularities of each behavioral addiction when assessing suicidal risk. The current study may contribute to better understanding of the suicidal phenomenon in this profile of patients and has clear clinical and research implications to improve prevention and treatment programs.

## Figures and Tables

**Table 1:** Descriptive for the sample.

			Gambling (*n* = 4103)		Gaming (*n* = 99)		Sex (*n* = 44)		Buying (*n* = 158)		
			*n*	*%*	*n*	*%*	*n*	*%*	*n*	*%*	*p*
Gender	Women		361	8.8%	7	7.1%	1	2.3%	118	74.7%	<0.001 *
	Men		3742	91.2%	92	92.9%	43	97.7%	40	25.3%	
Marital	Single		1676	40.8%	90	90.9%	12	27.3%	62	39.2%	<0.001 *
	Married/ Partner		1876	45.7%	8	8.1%	24	54.5%	72	45.6%	
	Divorced/ Separated		551	13.4%	1	1.0%	8	18.2%	24	15.2%	
Social	Mean-high to high		248	6.0%	4	4.0%	10	22.7%	27	17.1%	<0.001 *
	Mean		428	10.4%	13	13.1%	6	13.6%	20	12.7%	
	Mean-low		1304	31.8%	26	26.3%	15	34.1%	45	28.5%	
	Low		2123	51.7%	56	56.6%	13	29.5%	66	41.8%	
Education	Primary		2399	58.5%	49	49.5%	12	27.3%	60	38.0%	<0.001 *
	Secondary		1449	35.3%	41	41.4%	19	43.2%	64	40.5%	
	University		255	6.2%	9	9.1%	13	29.5%	34	21.5%	
Occupational	Unemployment		1797	43.8%	79	79.8%	13	29.5%	83	52.5%	<0.001 *
	Employed		2306	56.2%	20	20.2%	31	70.5%	75	47.5%	
Family support	No/partial		1014	24.7%	11	11.1%	16	36.4%	55	34.8%	<0.001 *
	Yes		3089	75.3%	88	88.9%	28	63.6%	103	65.2%	
Debts due to beh.add.	No		765	18.6%	70	70.7%	30	68.2%	48	30.4%	<0.001 *
	Yes		3338	81.4%	29	29.3%	14	31.8%	110	69.6%	
		α	*Mean*	*SD*	*Mean*	*SD*	*Mean*	*SD*	*Mean*	*SD*	*p*
Chronological age (years-old)			42.46	13.77	23.03	9.73	43.07	11.81	43.43	11.52	<0.001 *
Onset of disorder (years-old)			30.23	12.11	18.40	8.74	32.89	12.81	33.75	12.17	<0.001 *
Duration of disorder (years)			6.10	6.08	3.99	2.84	6.34	5.66	7.03	6.20	0.001 *
SCL-90R Somatic		0.908	0.96	0.79	0.63	0.66	1.01	0.82	1.46	1.02	<0.001 *
SCL-90R Obsessive-compulsive		0.884	1.15	0.80	1.08	0.76	1.48	0.99	1.87	1.01	<0.001 *
SCL-90R Interpersonal sensit.		0.876	1.03	0.81	1.11	0.97	1.31	0.88	1.58	1.05	<0.001 *
SCL-90R Depression		0.913	1.51	0.89	1.06	0.91	1.68	0.97	2.11	1.04	<0.001 *
SCL-90R Anxiety		0.897	1.02	0.80	0.70	0.71	1.20	0.93	1.56	1.06	<0.001 *
SCL-90R Hostility		0.853	0.92	0.82	0.96	0.88	1.16	1.01	1.33	1.04	<0.001 *
SCL-90R Phobic anxiety		0.829	0.49	0.66	0.40	0.66	0.53	0.69	0.96	1.05	<0.001 *
SCL-90R Paranoia		0.789	0.93	0.77	1.01	0.92	1.14	0.85	1.38	0.97	<0.001 *
SCL-90R Psychotic		0.860	0.91	0.75	0.64	0.71	1.33	0.88	1.17	0.88	<0.001 *
SCL-90R GSI		0.981	1.06	0.69	0.86	0.68	1.27	0.78	1.57	0.88	<0.001 *
SCL-90R PST		0.981	46.18	21.69	38.02	20.38	51.18	23.25	55.77	21.07	<0.001 *
SCL-90R PSDI		0.981	1.89	0.59	1.81	0.62	2.11	0.59	2.35	0.68	<0.001 *
TCI-R Novelty seeking		0.702	109.24	13.12	102.19	12.93	107.34	14.84	114.05	14.01	<0.001 *
TCI-R Harm avoidance		0.818	100.81	15.81	102.26	18.69	102.70	16.10	110.89	18.75	<0.001 *
TCI-R Reward dependence		0.772	98.16	13.61	93.32	17.03	97.91	16.20	101.98	15.40	<0.001 *
TCI-R Persistence		0.886	108.23	18.50	94.75	18.53	104.36	20.52	106.37	17.03	<0.001 *
TCI-R Self-directedness		0.856	127.48	19.87	128.30	22.03	120.16	20.21	123.89	22.07	0.013 *
TCI-R Cooperativeness		0.806	130.22	15.13	129.47	18.47	124.93	16.17	132.91	15.29	0.015 *
TCI-R Self-transcendence		0.831	63.47	14.07	59.82	14.83	63.32	14.40	65.54	15.32	0.019 *

Note. SD: standard deviation. α: Cronbach alpha in the sample. * Bold: significant comparison (0.05).

**Table 2:** Comparison between the groups with and without suicidal behavior (ideation or attempts).

		Gambling Disorder						Gaming Disorder						Sex Addiction						Buying–Shopping Disorder					
		SB-; *n* = 3163		SB+; *n* = 940				SB-; *n* = 93		SB+; *n* = 6				SB-; *n* = 36		SB+; *n* = 8				SB-; *n* = 129		SB+; *n* = 29			
		*n*	*%*	*n*	*%*	*p*	*|d|*	*n*	*%*	*n*	*%*	*p*	*|d|*	*n*	*%*	*n*	*%*	*p*	*|d|*	*n*	*%*	*n*	*%*	*p*	*|d|*
Gender	Women	248	7.8%	113	12.0%	0.001 *	0.14	5	5.4%	2	33.3%	0.010 *	**0.76** **^†^**	1	2.8%	0	0.0%	0.633	0.33	91	70.5%	27	93.1%	0.012 *	**0.62** **^†^**
	Men	2915	92.2%	827	88.0%			88	94.6%	4	66.7%			35	97.2%	8	100%			38	29.5%	2	6.9%		
Marital	Single	1315	41.6%	361	38.4%	0.001 *	0.06	85	91.4%	5	83.3%	0.709	0.25	10	27.8%	2	25.0%	0.280	0.06	54	41.9%	8	27.6%	0.206	0.30
	Married/Partner	1465	46.3%	411	43.7%		0.05	7	7.5%	1	16.7%		0.29	21	58.3%	3	37.5%		0.42	58	45.0%	14	48.3%		0.07
	Divorced/Separated	383	12.1%	168	17.9%		0.16	1	1.1%	0	0.0%		0.21	5	13.9%	3	37.5%		**0.55** **^†^**	17	13.2%	7	24.1%		0.28
Social	Mean-high to high	193	6.1%	55	5.9%	0.492	0.01	4	4.3%	0	0.0%	0.546	0.42	8	22.2%	2	25.0%	0.431	0.07	25	19.4%	2	6.9%	0.010 *	0.38
	Mean	333	10.5%	95	10.1%		0.01	13	14.0%	0	0.0%		**0.77** **^†^**	5	13.9%	1	12.5%		0.04	19	14.7%	1	3.4%		0.41
	Mean-low	1021	32.3%	283	30.1%		0.05	25	26.9%	1	16.7%		0.25	14	38.9%	1	12.5%		**0.62** **^†^**	30	23.3%	15	51.7%		**0.60** **^†^**
	Low	1616	51.1%	507	53.9%		0.06	51	54.8%	5	83.3%		**0.63** **^†^**	9	25.0%	4	50.0%		**0.52** **^†^**	55	42.6%	11	37.9%		0.10
Education	Primary	1845	58.3%	554	58.9%	0.909	0.01	45	48.4%	4	66.7%	0.421	0.37	11	30.6%	1	12.5%	0.578	0.45	49	38.0%	11	37.9%	0.991	0.00
	Secondary	1119	35.4%	330	35.1%		0.01	40	43.0%	1	16.7%		**0.59** **^†^**	15	41.7%	4	50.0%		0.17	52	40.3%	12	41.4%		0.02
	University	199	6.3%	56	6.0%		0.01	8	8.6%	1	16.7%		0.25	10	27.8%	3	37.5%		0.21	28	21.7%	6	20.7%		0.02
Occupational	Unemployment	1358	42.9%	439	46.7%	0.041 *	0.08	73	78.5%	6	100%	0.204	**0.96** **^†^**	11	30.6%	2	25.0%	0.755	0.12	62	48.1%	21	72.4%	0.018 *	**0.50** **^†^**
	Employed	1805	57.1%	501	53.3%			20	21.5%	0	0.0%			25	69.4%	6	75.0%			67	51.9%	8	27.6%		
Family Support	No	742	23.5%	272	28.9%	0.001*	0.12	10	10.8%	1	16.7%	0.655	0.19	11	30.6%	5	62.5%	.089	**0.65** **^†^**	39	30.2%	16	55.2%	0.011 *	**0.51** **^†^**
	Yes	2421	76.5%	668	71.1%			83	89.2%	5	83.3%			25	69.4%	3	37.5%			90	69.8%	13	44.8%		
Debts	No	639	20.2%	126	13.4%	0.001 *	0.18	66	71.0%	4	66.7%	0.822	0.17	25	69.4%	5	62.5%	0.703	0.15	41	31.8%	7	24.1%	0.419	0.17
	Yes	2524	79.8%	814	86.6%			27	29.0%	2	33.3%			11	30.6%	3	37.5%			88	68.2%	22	75.9%		
		*Mean*	*SD*	*Mean*	*SD*	*p*	*|d|*	*Mean*	*SD*	*Mean*	*SD*	*p*	*|d|*	*Mean*	*SD*	*Mean*	*SD*	*p*	*|d|*	*Mean*	*SD*	*Mean*	*SD*	*p*	*|d|*
Age (yrs)		42.1	14.1	43.8	12.4	0.001 *	0.13	22.7	9.2	28.8	16.5	0.132	0.46	43.1	12.0	42.8	11.5	0.934	0.03	43.7	12.0	42.3	9.3	0.576	0.12
Onset disorder (yrs)		30.1	12.3	30.7	11.4	0.213	0.05	18.3	8.4	20.5	13.8	0.546	0.20	32.4	13.2	35.3	11.1	0.570	0.24	33.6	12.5	34.4	11.0	0.759	0.07
Duration disorder (yrs)		6.0	6.0	6.5	6.4	0.014 *	0.09	3.9	2.9	5.0	1.8	0.371	0.45	6.9	5.9	3.9	3.7	0.176	**0.61** **^†^**	7.2	6.3	6.4	5.6	0.554	0.13
SCL-90R Somatic		0.9	0.7	1.2	0.9	0.001 *	0.41	0.6	0.6	0.8	1.0	0.459	0.25	1.0	0.9	1.2	0.6	0.474	0.32	1.3	1.0	2.0	1.0	0.001 *	**0.67** **^†^**
SCL-90R Ob-compulsive		1.1	0.8	1.4	0.8	0.001 *	0.46	1.0	0.7	1.6	0.9	0.042 *	**0.75** **^†^**	1.5	1.0	1.3	0.7	0.536	0.28	1.8	1.0	2.4	0.9	0.002 *	**0.66** **^†^**
SCL-90R Inter.sens.		1.0	0.8	1.3	0.9	0.001 *	0.38	1.1	0.9	1.3	1.2	0.584	0.19	1.4	0.9	1.1	0.7	0.449	0.33	1.5	1.0	2.1	1.1	0.003 *	**0.61** **^†^**
SCL-90R Depression		1.4	0.8	1.9	0.9	0.001 *	**0.63** **^†^**	1.1	0.9	1.2	0.8	0.611	0.22	1.7	1.0	1.5	0.5	0.640	0.22	2.0	1.0	2.8	0.8	0.001 *	**0.84** **^†^**
SCL-90R Anxiety		0.9	0.7	1.3	0.9	0.001 *	**0.51** **^†^**	0.7	0.7	1.2	1.1	0.045 *	**0.57** **^†^**	1.2	1.0	1.0	0.6	0.606	0.24	1.4	1.0	2.2	1.1	0.001 *	**0.76** **^†^**
SCL-90R Hostility		0.9	0.8	1.1	0.9	0.001 *	0.33	0.9	0.9	1.5	0.9	0.140	**0.64** **^†^**	1.2	1.1	0.9	0.7	0.444	0.33	1.2	1.0	1.9	1.1	0.001 *	**0.72** **^†^**
SCL-90R Phobic anxiety		0.4	0.6	0.7	0.8	0.001 *	0.33	0.4	0.6	0.6	0.9	0.508	0.24	0.6	0.7	0.3	0.4	0.379	0.41	0.8	0.9	1.6	1.3	0.001 *	**0.69** **^†^**
SCL-90R Paranoia		0.9	0.7	1.1	0.8	0.001 *	0.33	1.0	0.9	1.4	1.3	0.230	0.43	1.1	0.9	1.2	0.8	0.955	0.02	1.2	0.9	2.0	1.0	0.001 *	**0.78** **^†^**
SCL-90R Psychotic		0.8	0.7	1.2	0.8	0.001 *	0.45	0.6	0.7	0.9	0.7	0.443	0.32	1.3	0.9	1.3	0.7	0.790	0.11	1.0	0.8	1.8	0.9	0.001 *	**0.87** **^†^**
SCL-90R GSI		1.0	0.7	1.3	0.7	0.001 *	**0.53** **^†^**	0.8	0.7	1.2	0.8	0.216	**0.52** **^†^**	1.3	0.8	1.2	0.5	0.735	0.15	1.4	0.8	2.2	0.8	0.001 *	**0.88** **^†^**
SCL-90R PST		44.1	21.7	53.3	20.2	0.001 *	**0.51** **^†^**	37.3	19.7	48.6	23.4	0.175	**0.52** **^†^**	50.7	24.2	53.4	19.7	0.766	0.13	53.1	21.0	66.8	17.4	0.002 *	**0.71** **^†^**
SCL-90R PSDI		1.8	0.6	2.1	0.6	0.0010*	**0.52** **^†^**	1.8	0.6	2.0	0.5	0.532	0.29	2.1	0.6	2.0	0.5	0.554	0.25	2.3	0.7	2.8	0.6	0.001 *	**0.75** **^†^**
TCI-R Novelty seeking		108.9	13.3	110.5	12.6	0.005 *	0.13	102.0	13.2	105.6	7.3	0.528	0.34	107.1	15.9	108.6	9.4	0.799	0.12	113.1	14.2	117.7	13.2	0.118	0.33
TCI-R Harm avoidance		99.6	15.6	104.9	15.7	0.001 *	0.34	101.8	18.2	108.7	23.6	.389	0.33	103.3	17.4	99.8	7.7	0.589	0.26	111.0	18.9	111.0	18.5	0.986	0.00
TCI-R Reward depend.		98.4	13.7	97.2	13.4	0.013 *	0.09	93.0	17.1	98.6	16.9	.457	0.33	97.9	16.8	98.2	14.4	0.962	0.02	103.2	15.1	97.2	16.4	0.062	0.38
TCI-R Persistence		108.5	18.6	107.2	18.1	0.046 *	0.07	95.2	18.1	87.9	25.6	.375	0.33	104.3	19.9	104.7	24.7	0.958	0.02	106.1	17.2	107.4	16.6	0.714	0.08
TCI-R Self-directedness		129.3	19.9	121.4	18.3	0.001 *	0.41	128.9	21.9	119.0	21.3	.295	**0.51** **^†^**	118.0	20.0	130.0	19.9	0.130	**0.60** **^†^**	126.2	21.5	113.7	22.6	0.007 *	**0.57** **^†^**
TCI-R Cooperativeness		130.8	15.2	128.2	14.9	0.001 *	0.17	129.8	18.5	124.8	19.6	.544	0.26	123.5	16.1	131.2	16.3	0.215	0.47	134.7	14.6	125.4	17.0	0.004 *	**0.59** **^†^**
TCI-R Self-transcend.		63.0	14.1	65.2	13.8	0.001 *	0.16	59.2	14.0	69.7	22.3	.098	**0.56** **^†^**	64.7	14.3	56.9	13.6	0.154	**0.56** **^†^**	64.3	15.1	70.3	15.6	.054	0.39

Note. SB-: without suicidal behavior. SB+: with suicidal behavior. SD: standard deviation. * Bold: significant comparison (0.05). ^†^ Bold: effect size into the mild–moderate (|*d*| > 0.50) to the high–large range (|*d*| > 0.80). Comparisons for the psychopathological state (SCL-90R) and the personality traits (TCI-R) adjusted by sex and age.

## Data Availability

The data presented in this study is available on request from the corresponding author.
